# Altered interhemispheric functional connectivity in remitted bipolar disorder: A Resting State fMRI Study

**DOI:** 10.1038/s41598-017-04937-6

**Published:** 2017-07-05

**Authors:** Lianping Zhao, Ying Wang, Yanbin Jia, Shuming Zhong, Yao Sun, Zhangzhang Qi, Zhongping Zhang, Li Huang

**Affiliations:** 1 Medical Imaging Center, First Affiliated Hospital of Jinan University, Guangzhou, 510630 China; 2 Department of Radiology, Gansu Provincial Hospital, Gansu, 730000 China; 3 Clinical Experimental Center, First Affiliated Hospital of Jinan University, Guangzhou, 510630 China; 4 Department of Psychiatry, First Affiliated Hospital of Jinan University, Guangzhou, 510630 China; 5 General Electric Healthcare, Shanghai, 200000 China

## Abstract

Abnormalities in structural and functional brain connectivity have been increasingly reported in patients with bipolar disorder (BD). However, alterations of remitted BD (RBD) in functional connectivity between the cerebral hemispheres are still not well understood. This study was designed to analyze the pattern of the interhemispheric functional connectivity of the whole brain in patients with remitted BD during resting state. Twenty patients with RBD and 38 healthy controls (HC) underwent the resting-state functional magnetic resonance imaging. The functional connectivity between any pair of symmetrical interhemispheric voxels (i.e., functional homotopy) was measured by voxel-mirrored homotopic connectivity (VMHC). The patients with RBD showed lower VMHC than HC in the middle frontal gyrus and precentral gyrus. No regions of increased VMHC were detected in the RBD patients. There were no significant correlations between the VMHC values in these regions and the clinical variables. These findings suggest substantial impairment of interhemispheric coordination in RBD and they may represent trait, rather than state, neurobiological feature of brain function in BD.

## Introduction

Bipolar disorder (BD) is a chronic, severe, and fluctuating mental disorder and the worldwide prevalence of the BD spectrum is 1–4%^1^. The psychiatric condition of BD commonly characterized by cyclically alternating depressive and manic episodes interspersed with symptomatic remissions. Symptomatic remissions are not free of residual cognitive and/or emotional symptoms^2, 3^. Some of these symptoms actually represent trait characteristics of the illness. Although researchers agree that BD is multifactorial with inherited, genetic and environmental risk factors, the neuropathological mechanisms are still remains unclear. The neuropathological changes of remitted BD (RBD)are most close to the pathophysiological determinants in that it may elucidate potential neurobiological mechanisms of BD while mitigating the possible confounding effects of current mood state, illness severity, nonspecific effects of chronic illness and stress. Consequently, identification of neuropathological alterations in RBD may contribute to early diagnosis of BD thus reducing the latency to adequate treatment and improving out-come.

Evidences from literatures have revealed that the imbalance between left and right hemispheric activity may contribute to the pathophysiology of BD^4, 5^. The corpus callosum (CC), the largest white matter structure connecting the left and right cerebral hemispheres, plays a pivotal role in interhemispheric communication and coordination, especially in the integration of emotional, cognitive, motor, and sensory information. Structural magnetic resonance imaging (MRI) studies have found area and shape changes in the CC in BD^6^. In addition, diffusion tensor imaging (DTI) and tractography studies have found reduced fractional anisotropy (FA) in the genu, body, and splenium of the CC in patients with BD during mood episodes^7, 8^ and in euthymic phase^9, 10^ relative to controls, suggesting that disruption of the organization of fiber tracts may be related to efficiency of interhemispheric signal transfer in the case of the CC. A recent study reported impaired interhemispheric but relatively preserved intrahemispheric integration in BD using DTI and graph theoretical analysis^11^. However, relatively little knows about the alterations in functional connectivity between the bilateral cerebral hemispheres in patients with RBD.

Resting-state fMRI (rs-fMRI), which reveals the patterns of coherent spontaneous fluctuations of blood oxygen level dependent (BOLD) signals^12^, provides fresh insights intra-and interhemispheric functional connectivity. The widely used approaches of rs-fMRI conducted in BD have employed either seed-based or amplitude of low-frequency fluctuation (ALFF) analyses^13^. While informative, the seed-based approach is always limited by the inherent bias introduced by predefining regions of interest (the seed region) for connectivity analyses. By contrast, ALFF is a useful tool for identifying between group differences in local BOLD signal frequency fluctuations, but is not a direct measure of functional connectivity. Kenny *et al*.^14^ have also emphasized that affective disorders are more likely due to aberrations at the circuit level rather than at a localized brain region. Functional homotopy is designed to quantify the high degree of synchrony in spontaneous activity between geometrically corresponding interhemispheric counterparts, one of the most salient features of the brain intrinsic functional architecture^15^, likely reflecting the importance of interhemispheric communication to integrated brain function underlying coherent cognition and behavior^16^. Voxel-mirrored homotopic connectivity (VMHC) is a recently validated approach which quantifies the resting-state functional connectivity between each voxel in one hemisphere and its corresponding voxel in the opposite hemisphere^17^. Recent work has demonstrated that VMHC is a reliable and reproducible rs-fMRI metric^18^. Since VMHC first introduced by Zuo *et al*.^17^, it has revealed aberrant interhemispheric functional connectivity in several neuropsychiatric disorders including schizophrenia^19, 20^, cocaine addiction^16^, migraine^21^, somatization disorder^22^, post-traumatic stress disorder^23^ and major depressive disorder (MDD)^24–27^. Our previous study revealed reduced interhemispheric resting-state functional connectivity in the medial prefrontal cortex and inferior temporal gyrus in unmedicated BD II depression^5^. However, there are no studies to investigate possible impairment of homotopic resting-state functional connectivity in RBD patients so far.

In the present study, we compared the interhemispheric resting-state functional connectivity in patients with RBD and healthy controls (HC) by use of VMHC analysis. Reduced VMHC values in patients with RBD were anticipated, which would suggest an impairment of interhemispheric functional coordination. We believe such data will contribute to our improved understanding of the pathophysiology of BD.

## Material and Methods

### Subjects

A cohort of 24 right-handed, out- or in-patients with RBD (23 remitted from BD-II, 1 remitted from BD-I) were recruited from the psychiatry department of First Affiliated Hospital of Jinan University, Guangzhou, China. The patients were aged from 18 to 47 years. All patients met DSM-IV criteria for BD-I or II in past diagnosis according to the diagnostic assessment by the Structured Clinical Interview for DSM-IV Patient Edition (SCID-P). At the time of the measurements all patients were in disease remission. Remitted state was defined by Hamilton Rating Scale for Depression-24 (HAMD-24) scores of <8 and Young Mania Rating Scale (YMRS) scores of <6, and the depressive or manic symptoms disappeared for at least or more than 6 months. At the time of the study, 4 patients in the BD group were without medication and 20 were receiving antidepressants (duloxetine or paroxetine), and/or mood stabilizers (lithium, sodium valproate), and/or atypical antipsychotic medications (olanzapine, risperidone orquetiapine). Exclusion criteria included the presence of (i) any current psychiatric disorder (with the exception of BD and anxiety disorders), (ii) a history of electroconvulsive therapy, (iii) any history of moderate/severe head injury, head trauma, neurological disorder, or mental retardation, (iv) alcohol/substance abuse or dependence, (v) the presence of a concurrent significant physical illness, and (vi) pregnancy or any contraindication to MRI scanning. A total of 42 right-handed age and gender matched healthy control subjects were recruited via local advertisements. They were carefully screened through a diagnostic interview, the Structured Clinical Interview for DSM-IV Nonpatient Edition (SCID-NP), to rule out the presence of current or past psychiatric illness. Further exclusion criteria for HC were any history of psychiatric illness in first-degree relatives, current or past significant medical or neurological illness. The study was approved by the Ethics Committee of First Affiliated Hospital of Jinan University (China) and the methods and procedures were carried out in accordance with the approved guidelines. All subjects signed a written informed consent form after a full written and verbal explanation of the study. Two senior clinical psychiatrists confirmed that all subjects had the ability to consent to participate in the examination.

### MRI data acquisition and preprocessing

MRI data were acquired on a 3.0 T MR system (Discovery MR 750 System, GE Healthcare, Milwaukee, WI) with an 8-channel phased array head coil. Subjects were scanned in a supine, head-first position with symmetrically placed cushions on both sides of the head to decrease motion. During the scanning, participants were instructed to relax with their eyes closed without falling asleep; after the experiment, each participant confirmed not having fallen asleep. The rs-fMRI data were acquired using gradient echo echo-planar imaging sequence with the following parameters: time repetition (TR)/time echo (TE) = 2000/25 ms, slice thickness/gap = 3.0/1.0 mm, voxel size = 3.75 × 3.75 × 3 mm^3^, field of view (FOV) = 240 × 240 mm, flip angle = 90°, matrix = 64 × 64, number of slices = 35, and NEX = 1. A 7-min scan (210 volumes) was obtained for each subject. In addition, a three dimensional brain volume imaging (3D BRAVO) sequence covering the whole brain was used for structural data acquisition with: TR/TE = 8.2/3.2 ms, slice thickness/gap = 1.0/0 mm, matrix = 256 × 256, FOV = 240 × 240 mm, NEX = 1, flip angle = 12°, bandwidth = 31.25 Hz, and acquisition time = 3 min 45 s. Routine MRI examination images were also collected for excluding anatomic abnormality, such as T1, T2, DWI, and T2- FLAIR images. All participants were confirmed by two experienced radiologists to have no abnormalities on routine MRI.

### Functional image preprocessing

The preprocessing was carried out using Data Processing Assistant for Resting-State fMRI (DPARSF)^28^ which is based on Statistical Parametric Mapping (SPM8, http://www.fil.ion.ucl.ac.uk/spm) and rs-fMRI Data Analysis Toolkit (REST, http://www.restfmri.net)^29^. For each individual, the first 10 images were discarded to ensure steady-state longitudinal magnetization. The remaining images were slice-time corrected and realigned to the first image in the first series. We controlled for head motion using a threshold of 1.0 mm translation in any cardinal direction and 1.0° rotation in each of the orthogonal x, y and z axes. The individual T1 images were coregistered to the mean of the realigned EPI images. The coregistered T1 structural images were then segmented (gray matter, white matter, and cerebrospinal fluid) using the unified segmentation algorithm and were then transformed from the individual native space to standard Montreal Neurological Institute (MNI) space. The functional images were also spatially normalized to MNI space by applying the parameters of structural image normalization and were resampled to 3 × 3 × 3 mm^3^ resolution. The generated images were spatially smoothed with a Gaussian kernel of 4 mm at full width at half-maximum. The resulting fMRI data were linearly trend removed and band-pass (0.01–0.08 Hz) filtered to reduce low-frequency drift and high-frequency noise. Resting-state fMRI measures are sensitive to micro-head motions^30^, therefore, the Friston 24-Parameter Model^30, 31^ was used to regress head motion effects from the realigned data (the 24 parameters include six head motion parameters, six head motion parameters one time point before, and the 12 corresponding squared items), based on recent reports demonstrating that higher-order models benefit from the removal of head motion effects^30, 31^. We further characterized the mean frame-wise displacement (FD), which considers measures of voxel-wise differences in motion in its derivation^32^, as a measure of the micro-head motion of each subject. Several nuisance variables including mean signals from white matter and cerebrospinal fluid, as well as the mean time series of all voxels across the whole brain, were removed from the preprocessed time-courses by multiple linear regression analysis.

### Voxel-mirrored homotopic connectivity

VMHC analysis was performed using DPARSF software. A left-right hemisphere symmetric brain template was generated from the all subjects to minimize the effects of geometric differences between the two hemispheres according to Zuo *et al*.’s method^17^. First, a mean normalized T1 image was generated by averaging all the spatially normalized T1 images. Next, the group-specific symmetrical template was obtained by averaging the mean normalized T1 image and its left-right mirrored version (flipped over x axis). Then each individual T1 image that had been normalized to MNI space was co-registered nonlinearly to this group-specific symmetric template. The same transformation was then applied to the resting-state functional images. For each subject, the homotopic resting state functional connectivity was computed as the Pearson correlation (Fisher Z-transformed) between each voxel’s residual time series and that of its mirrored interhemispheric counterpart. The resultant values generated the VMHC maps and were used for subsequent group-level analyses. We also calculated the global VMHC for each subject and compared it between groups. Global VMHC was calculated by averaging VMHC values across all brain voxels.

### Statistical analysis

Independent-sample *t*-tests and chi-squared tests were used to compare demographic data between the RBD and HC groups with SPSS 17.0 software (SPSS, Chicago, IL, USA). All tests were two-tailed, and *P* < 0.05 was considered statistically significant. Nonparametric permutation tests (5,000 permutations) were used to detect statistically significant differences in VMHC between patients with RBD and controls^33^. Individual’s age, sex, and mean FD were used as nuisance covariates. Multiple comparisons were corrected using threshold-free cluster enhancement (TFCE) (*P* < 0.05)^34^, which allowed us to avoid arbitrarily choosing a cluster-forming threshold while enabling us to preserve the sensitivity benefits of the cluster-wise correction. Once significant group differences were observed in any brain regions, we further computed Pearson’s correlation coefficients between these VMHC values and the clinical variables including HAMD-24 score, YMRS score, age of illness onset, duration of total illness, duration of symptoms, duration of remissions (*p* < 0.05, Bonferroni corrected), and Spearman’s correlation coefficients between VMHC values and number of episodes (*p* < 0.05, Bonferroni corrected).

## Results

### Sample characteristics

Table 1 shows the demographic and clinical data of all study participants. Four patients with RBD and four HC were excluded from further analyses due to excessive head motion during the image acquisition. Finally, the participants included 20 patients with RBD and 38 HC subjects. There were no significant differences in sex, age and years of education between the RBD group and the HC group.

**Table 1:** The demographic characteristics of the subjects.

	RBD	HC	Statistic	
Number of subjects	20	38		
Gender (male/female)	7/13	13/25	χ^2^ = 0.004	*P* = 0.952
Age (years)	28.70 (10.18)	28.79 (10.18)	*t* = −0.032	*P* = 0.975
Education (years)	13.28 (2.65)	14.76 (3.13)	*t* = −1.737	*P* = 0.09
Age of illness Onset (years)	21.32 (6.61)	n/a	n	n
Duration of total illness (months)	59.50 (51.58)	n/a	n	n
Duration of symptoms (months)	43.64 (48.57)	n/a	n	n
Duration of remissions (months)	15.86 (18.87)	n/a	n	n
Number of episodes	3.73 (1.08)	n/a	n	n
HAMD-24 score (points)	2.59 (1.84)	n/a	n	n
YMRS score (points)	0.55 (1.30)	n/a	n	n
family history (yes/no)	4/20	n/a	n	n
FD values (mm)	0.09 (0.05)	0.06 (0.02)	*t* = 3.687	*P* = 0.005

Values are reported as mean (standard deviation), except for gender and family history. RBD = remitted bipolar disorder. HC = healthy controls. HAMD-24 = Hamilton Rating Scale for Depression-24. YMRS = Young Mania Rating Scale. FD = framewise displacement for in scanner head motion.

### VMHC: group differences

**Figure 1:**
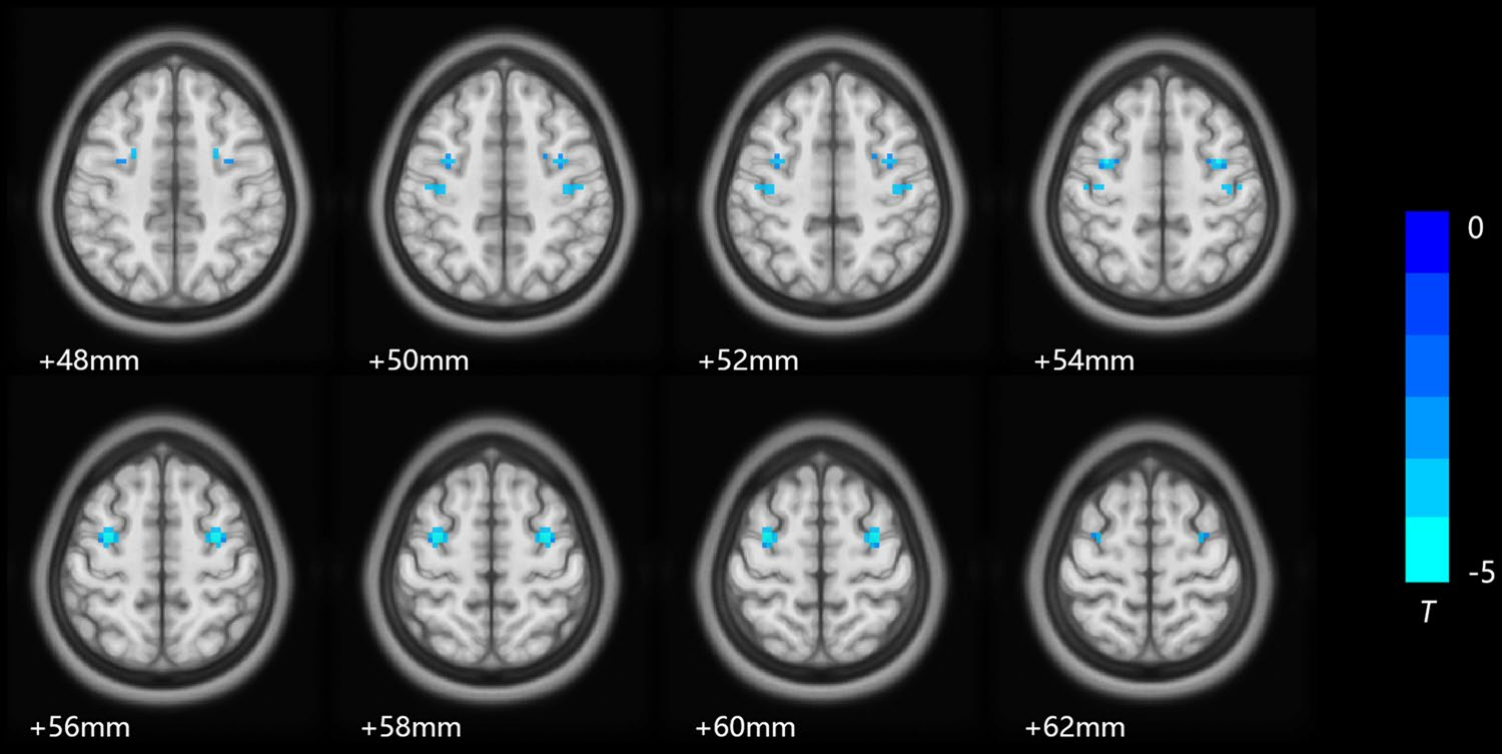
Axial MR images show significant differences in VMHC between patients with RBD and HC. Blue colors indicate reduced VMHC in RBD, and the color bar indicates the *T* value from *t*-test between groups.

The RBD patient group showed lower VMHC values than the control group in the middle frontal gyrus and precentral gyrus (Fig. 1 and Table 2). No region showed greater VMHC in the patient group than in the control group. There were no significant correlations between the VMHC values in these regions and any clinical measures (including HAMD-24 score, YMRS score, age of illness onset, duration of total illness, duration of symptoms, duration of remissions and number of episodes) after bonferroni multiple comparison correction.

**Table 2:** The areas of VMHC differences between patients with RBD and HC.

Brain regions	Brodman Area	Montreal Neurological institute Coordinates			Peak	Cluster
		X	Y	Z	*t* Value	Size (mm^3^)
Middle frontal gyrus	6	±33	−6	57	−5.353	1026
Precentral gyrus	4	±36	−18	51	−4.450	270

VMHC = voxel-mirrored homotopic connectivity. RBD = remitted bipolar disorder. HC = healthy controls.

## Discussion

The VMHC method adopted here to identify brain regions with abnormal connectivity has proved to be informative. Here, VMHC based on the rs-fMRI data was applied for the first time to investigate interhemispheric functional connectivity in patients with RBD. The main finding of this study was the significant decrease in the homologous resting-state functional connectivity in the brain regions of middle frontal gyrus and precentral gyrus in the patients with RBD.

In the current study, we observed decreased VMHC values in the middle frontal gyrus in RBD compared to controls. An rs-fMRI study also found lower VMHC in the medial prefrontal cortex in major depressive disorder^35^. The middle frontal gyrus is within the prefrontal lobe and plays an important role in the prefrontal-limbic system. The prefrontal lobe is generally considered to be the advanced cognitive center which participates in integrating emotion and information about internal and external environment, and extracting episodic memory in the resting-state^36^. Two rs-fMRI studies^36, 37^ revealed abnormal regional homogeneity in the middle frontal gyrus in BD in the depressive episode. Several researches using task-based fMRI, such as the modified word-based memory task, working memory, response inhibition, and emotional face encoding, indicated decreased or absent^38–42^ activity of the middle frontal gyrus and limbic system in BD patients and increased activity in pediatric BD after pharmacotherapy during a response inhibition task fMRI^43^. Several structural MRI^44–47^ also showed the smaller volumes or decreased gray matter in the right middle frontal gyrus in BD. Furthermore, it is interestingly that two of those studies^46, 47^ revealed a consistent result which showed only the volume of the right middle frontal gyrus was inversely correlated with illness duration in BD patients, indicating the middle frontal gyrus may play an important role in the onset of the illness. Studies from literature provide further structural disconnective evidence for functional connectivity abnormalities between the left and right hemispheres of the prefrontal lobe. An international multicenter study showed patients with BD-I had smaller adjusted mid-sagittal CC areas than controls along the posterior body, the isthmus and the splenium of the CC^48^. Additionally, a voxel-based meta-analysis revealed both major depressive disorder and BD are characterized by abnormalities in white matter tracts of the genu of the CC which connect the two hemispheres of the prefrontal cortex implicated in mood regulation^49^. Taken together, although these studies mentioned above were not conducted in homogeneous remitted episode of BD patients, in accordance with the present study, the results suggest reduced VMHC in the middle frontal gyrus may related to the impairment of emotional, cognitive, and sensory processing in patients with BD.

We observed decreased VMHC values in the precentral gyrus in RBD compared to controls, suggesting impaired functional connectivity in the precentral gyrus bilaterally. Precentral gyrus also belong to the prefrontal lobe. Evidences from literature^50, 51^ have indicated that the prefrontal lobe plays a key role in processing emotional information. The precentral gyrus consists of the primary motor cortex and is the cortical area responsible for voluntary movement^52, 53^, a systematic review^54^ indicated a general alteration in motor cortical inhibitionin mental illness, rather than disease-specific changes. However, It is also believed that the precentral gyrus is involved in cognitive processing and emotion regulation^55, 56^. It has been shown to activate in response to viewing^57^ or identifying^58, 59^ emotional face expressions. The activity in this region has been shown to be higher than healthy controls when viewing emotional faces in patient with schizophrenia^60^ and BD^55^. The precentral gyrus is also important for executive function, and increased precentral activity for BD vs. controls has been demonstrated in a working memory task^61^. Additionally, a resting-state fMRI study^62^ found youth with BD exhibited decreased connectivity between the laterobasal subdivision of the amygdala and the hippocampus and precentral gyrus, which supported our findings. Cantisani *et al*.^63^ found positive correlations of increased perfusion and reduced activity level in the left precentral gyrus in BD. Furthermore, numerous structural MRI^64–66^ demonstrated abnormalities in the precentral gyrus in BD, further suggesting the involvement of the precentral gyrus in the pathogenesis of BD. A study^64^ adopted vertex-wise cortical based brain imaging techniques showed BD patients had significantly larger cortical surface area in the left precentral gyrus compared to MDD patients. and a structural MRI^65^ by using Freesurfer technique found increased contrast between grey-and white-matter intensities in the left precentral gyrus in BD, indicating reduced intracortical myelin in this region. A VBM study^66^ found volume deficits in the left precentral gyrus of patients with BD compared to unrelated healthy controls. Intriguingly, van der Schot *et al*.^67^ also found the genetic risk to develop bipolar disorder was related to decreased grey matter density in the precentral gyrus by investigated 49 affected twin pairs and 67 healthy twin pairs, further suggesting the involvement of the precentral gyrus in the pathogenesis of BD. Therefore, the current study provides further evidence that dysfunction in emotion processing occurs not only in emotional regions of the brain, but also in the traditional motor contex–precentral gyrus.

The present study showed no significant correlations between the decreased VMHC brain regions and the clinical measures (including HAMD-24 score, YMRS score, age of illness onset, duration of total illness, duration of symptoms, duration of remissions and number of episodes). In a non-clinical depressive symptoms study^68^, significant correlations between altered ALFF values and the depressive severity scores (Beck Depression Inventory scores) was found. In their study, the mean Beck Depression Inventory scores in non-clinical depressive symptoms is 15.24, it met a moderate depression standard according to the Beck Depression Inventory. However, in our study, the mean HAMD-24score is 2.59 in RBD and this score does not meet any criteria for the depression diagnosis. Thus it’s not hard to understand there is no correlation between the altered VMHC regions and the HAMD-24 score or YMRS score in the RBD patients. it is possible that the alterations of VMHC brain regions (the middle frontal gyrus and precentral gyrus) may be a trait marker^24, 69^ for patients with RBD independent of the severity of symptoms and other related factors, as Guo, W. *et al*.^24^ reported there is no correlations between the VMHC in any brain region and the overall clinical variables in patients with MDD by voxel-based correlation analyses, and the author speculated that the VMHC abnormal brain regions represented trait characteristics of the disease. Bhagwagar *et al*.^69^ reported the biochemical abnormalities in recovered unmedicated BD and MDD may be markers of a trait vulnerability to mood disorder, rather than neurochemical correlates of an abnormal mood state. Nevertheless, longitudinal designed study and comparison with the symptomatic BD cohort is necessary to further confirm whether it is a trait marker for BD in future study.

The strengths of the study include the homogeneous samples of RBD. Although the findings of this study are promising, some limitations must be noted. First, the sample size of this study was relatively small, thus, the findings will require replication in a larger study. Second, most of the patients included were under medication prior to MRI scanning. Furthermore, the medication varied, and the number of subjects was too limited to group them according to a specific type of drug. Medication could play a specific role in brain spontaneous activity and functional connectivity but this has not been demonstrated. Studies using patients without medication would be useful for ruling out the effects of medication on the results. Third, the human brain is not symmetrical. However, we tried to solve this issue by applied a symmetrical template and smoothed the functional data to improve the functional correspondence between homotopic regions^17^. Hence morphometric asymmetry could not account for altered VMHC^16^. Also, it would be desirable to assess if reduced interhemispheric synchrony observed in patients with RBD is driven by brain activity in a single hemisphere. However, this is not feasible with this functional connectivity approach owing to the operational definition of VMHC analysis, which inheres that results are strictly symmetrical^27^. Additionally, the underlying mechanisms of altered homotopic connectivity in patients with RBD are largely unknown, it is possible that the structural changes may underlying abnormal interhemispheric synchrony include left-right asymmetries regarding homotopic gray matter volumes. However, Hermesdorf *et al*.’s^27^ investigated the reduced homotopic connectivity in MDD and the underlying structural mechanisms and reported no differences in left-right symmetries in homotopic gray matter volumes were evident across the cohorts. Thus future research that use a functional connectivity combined voxel-based morphometry, Freesurfer and DTI is necessary to clarify the unknown structural basis for VMHC alterations in RBD. Finally, we did not collect neuropsychological data, especially tasks that required interhemispheric transfer of information. Thus, additional neuropsychological tests are needed, and we will investigate the associations between cognitive dysfunction and VMHC deficits in further work.

## Conclusion

In conclusion, the current findings are the first to reveal aberrant interhemispheric functional connectivity in the bilateral middle frontal gyrus and precentral gyrus in patients with RBD. Since the disruption in functional coordination between the two hemispheres occurred in remission, they may represent trait, rather than state, neurobiological feature of brain function in bipolar disorder. Homotopic interhemispheric resting- state functional connectivity may provide a useful and sensitive screening approach for evaluating BD where neural connectivity is implicated in the pathophysiology.
